# Overexpression of *CpWRKY75* from *Chimonanthus praecox* Promotes Flowering Time in Transgenic Arabidopsis

**DOI:** 10.3390/genes13010068

**Published:** 2021-12-28

**Authors:** Renwei Huang, Shunzhao Sui, Huamin Liu, Mingyang Li, Daofeng Liu

**Affiliations:** 1 Sichuan Provincial Key Laboratory for Development and Utilization of Characteristic Horticultural Biological Resources, College of Chemistry and Life Sciences, Chengdu Normal University, Chengdu 611130, China; 081030@cdnu.edu.cn; 2 Chongqing Engineering Research Center for Floriculture, Key Laboratory of Horticulture Science for Southern Mountainous Regions of Ministry of Education, College of Horticulture and Landscape Architecture, Southwest University, Chongqing 400715, China; sszcq@swu.edu.cn (S.S.); limy@swu.edu.cn (M.L.); 3 College of Landscape Architecture and Life Science, Chongqing University of Arts and Sciences, Chongqing 402160, China; liuhuamin@cqwu.edu.cn

**Keywords:** wintersweet, *CpWRKY75*, expression pattern, flowering

## Abstract

WRKY transcription factors play critical roles in the physiological processes of plants. Although the roles of WRKYs have been characterized in some model plants, their roles in woody plants, especially wintersweet (*Chimonanthus praecox*), are largely unclear. In this study, a wintersweet WRKY gene named *CpWRKY75* belonging to group IIc was isolated and its characteristics were identified. CpWRKY75 is a nucleus-localized protein, and exhibited no transcriptional activation activity in yeast. *CpWRKY75* was highly expressed in flowers at different bloom stages. Ectopic expression of *CpWRKY75* significantly promoted the flowering time of transgenic Arabidopsis (*Arabidopsis thaliana*), as determined by the rosette leaf number and first flower open time. The expression levels of flowering-related genes were quantified by qRT-PCR, and the results suggested that *CpWRKY75* had obvious influence on the expression level of *MICRORNA156C* (*MIR156C*), *SQUAMOSA PROMOTER BINDING PROTEIN-LIKE* *3* (*SPL3*) and *SQUAMOSA PROMOTER BINDING PROTEIN-LIKE 9* (*SPL9*), *FLOWERING LOCUS T* (*FT*), *LEAFY* (*LFY*), *SUPPRESSOR OF OVEREXPRESSION OF CO 1* (*SOC1*), *APETALA1* (*AP1*), *CAULIFLOWER* (*CAL*), and *FRUITFULL* (*FUL*). These results suggest that *CpWRKY75* might have a flowering time regulation function, and additionally provide a new gene resource for the genetic engineering of woody flowering plants.

## 1. Introduction

Wintersweet (*Chimonanthus praecox*), also known as wax shrub, a deciduous shrub belonging to the *Calycanthaceae* family, is native to China and has been cultivated for over 1000 years. Wintersweet has bright yellow flowers with a sweet fragrance, and blooms from late November to March. Because of its particular flowering time and attractive fragrance, wintersweet is one of the most popular winter-season ornamental trees in China. It has great ornamental importance and can be used as a potted plant, cut flower and landscape plant [1]. However, as a kind of woody flowering plant the juvenile phase of wintersweet can last about 4–5 years [2], which severely impedes both expansion of commercial demand and breeding efficiency. Knowledge of the genes involved in flowering in model plants such as Arabidopsis (*Arabidopsis thaliana*) and identification of homologous genes in woody plants have provide new directions for early-flower induction [3]. Thus, understanding the regulatory mechanisms of flowering in wintersweet is critical for shortening the juvenile stage and breeding cycle. Nevertheless, the molecular mechanisms regulating wintersweet flowering are still largely unknown.

Flowering is a crucial phase for flowering plants which reflects the transition from vegetative growth to reproductive development [4]. The timing of flowering is determined by a complex interplay of endogenous cues and environmental signals [5]. Numerous genes governing flowering have been widely studied in various plants. In Arabidopsis, it has been reported that there are at least 180 genes relating to flowering-time control, in addition to those still-unknown or undetected genes [6]. Most of the flowering-related genes are transcriptional factors such as *AGAMOUS*-*LIKE 24* (*AGL24*), *APETALA1* (*AP1*), *CAULIFLOWER* (*CAL*), *FLOWERING LOCUS C* (*FLC*), *FRUITFULL* (*FUL*) and *SUPPRESSOR OF OVEREXPRESSION OF CO 1* (*SOC1*), which encode MADS-box transcription factors [7]. Besides these, members from other transcription factor families have also been found to play role in directly or indirectly regulating flowering. For instance, *AtPIF4*, which encodes a bHLH protein, is involved in regulating flowering by positively modulating *FT* expression [8]. MlNAC5, a NAC transcription factor isolated from *Miscanthus lutarioriparius*, delays flowering time in Arabidopsis [9]. AtKHZ1 and AtKHZ2, two CCCH family proteins, play redundant roles in regulating flowering [10].

WRKY proteins are plant-specific regulatory proteins. They contain one or two DNA binding domain known as WRKY, consisting of approximately 60 amino acids with a nearly constant sequence WRKYGQK at the N terminus and a C2H2 or C2HC zinc finger motif at the C terminus. To regulate the expression of target genes, the WRKY domain recognizes and binds specifically to the cis-element termed W-box [TTGAC(C/T)] in their promoters to modulate transcription. The family has been divided into three subfamilies based on the WRKY domain numbers and the features of the zinc finger motif. Group II members are further clustered into five subgroups (IIa-IIe) based on other structural motifs [11]. The WRKY protein was first isolated from sweet potato in 1994 [12]; subsequently, many WRKY genes have been cloned from various plant species such as Arabidopsis [13], rice (*Oryza sativa*) [14], grape *(Vitis vinifera*) [15] and soybean (*Glycine max*) [16] and reported to participate in various life processes in plants. Ectopic expression of *Lilium regale LrWRKY4* and *LrWRKY12* in Arabidopsis enhanced resistance to *Botrytis cinerea* [17]. In *Dendranthema grandiflorum* (chrysanthemum), *DgWRKY5* has been demonstrated to increase salt stress tolerance in transgenic chrysanthemum by up-regulating stress-related genes [18]. In cotton (*Gossypium hirsutum*), *GhWRKY42* and *GhWRKY91* were reported to function as positive and negative regulators of leaf senescence [19,20], respectively. In *Malus domestica*, MdWRKY11 was found to participate in anthocyanin accumulation by directly regulating *MdMYB10*, *MdMYB11*, *MdUFGT* and *MdHY5* [21]. In foxtail millet, *LP1* was identified as involved in the regulation of panicle development, seed size, and stem elongation [22]. In recent years, emerging evidence has demonstrated that WRKY proteins participate in flowering time regulation. For example, the rice *Dlf1* gene which encodes the OsWRKY11 protein was found to suppress flowering by repressing the transcription of *Ehd2*/*RID1*/*OsId1* under LD and SD conditions [23]. FvWRKY71 promotes flowering time by directly activating the expression of *FvFUL*, *FvSEP1*, *FvAGL42*, *FvLFY* and *FvFPF1* [24]. AtWRKY12 and AtWRKY13 play an opposite role in modulating flowering time by directly regulating *FUL* [25]. *GmWRKY58* and *GmWRKY76*, isolated from soybean, which were found to promote flowering in Arabidopsis [26]. At present, research into WRKY-controlled flowering has mainly concentrated on model and annual plants, while little is known about the role of WRKYs in flowering regulation of woody plants.

In this study, a novel WRKY gene from wintersweet named *CpWRKY75* was isolated. The characteristics of *CpWRKY75* were identified and its expression pattern was investigated. Meanwhile, overexpression of *CpWRKY75* was shown to promote flowering time of transgenic Arabidopsis. Our results could provide a new flowering time-related gene for wintersweet breeding.

## 2. Materials and Methods

### 2.1. Plant Materials

For gene expression analysis, wintersweet tissues (roots, stems, young leaves, flowers at sprout stage, flowers at petal-display stage, flowers at bloom stage) were collected. Roots, stems, and young leaves were harvested from six-leaf stage wintersweet [27]. Flowers at different bloom stages were collected from the adult plant growing in Southwest University as described by Sui et al. [28].

For subcellular localization, tobacco (*Nicotiana benthamiana*) seeds were directly sown in a mixture of peat and wormcast (2:1) and maintained at 25 °C in a growth chamber with a 16/8 h day/night photoperiod.

For plant transformation, seeds of Arabidopsis (ecotype Columbia) were surface-sterilized and cultured on solid Murashige and Skoog (MS). After 10 days, the seedlings were planted in the culturing bowl filled with peat and wormcast (2:1) and maintained in a growth chamber (22 °C, 16/8 h day/night photoperiod).

### 2.2. RNA Extraction and qRT-PCR Analysis

Trizol (Invitrogen, Carlsbad, CA, USA) was used for total RNA isolation according to the manufacture. Primescript RT reagent kit (Takara, Dalian, China) was used to synthesize cDNAs. qRT-PCR was performed using the Bio-Rad machine with the Ssofast EvaGreen Supermix (BIO-RAD, Hercules, CA, USA). *CpActin* and *CpTublin* were chose as the reference gene for wintersweet [28]. *AtActin* was chose as the reference gene for Arabidopsis [29]. Gene expression levels were calculated by comparative CT method [29]. qRT-PCR primers used in this study were described in Supplementary Table S1.

### 2.3. Cloning and Characterization of CpWRKY75

Full length cDNA and genomic sequence of *CpWRKY75* were amplified from wintersweet with CpWRKY75-F and CpWRKY75-R as primers (Supplementary Table S1). PCR fragments were purified and introduced into PMD19-T (Takara, Dalian, China), then introduced into *E. coli* strain TOP10 for the following sequencing (BGI, Shenzhen). Theoretical isoelectric point (pI) and molecular weight of CpWRKY75 were predicted by ExPASy (http://web.expasy.org/compute_pi/, accessed on 18 August 2021). Multiple Sequence Alignment was performed using Bioedit 7.0. A phylogenetic tree of CpWRKY75 and other WRKY protein was constructed by MEGA 11 (neighbor-joining method), and bootstrap analysis using 1000 replicates with the Poisson model and pairwise deletion [30]. WoLF PSORT (https://wolfpsort.hgc.jp/, accessed on 18 August 2021). and ProtComp (http://www.softberry.com/berry.phtml?topic=protcomppl&group=programs&subgroup=proloc, accessed on 18 August 2021). were adopted for subcellular localization prediction of CpWRKY75.

### 2.4. Subcellular Localization of CpWRKY75

The coding sequence (CDS) of *CpWRKY75* without stop codon was inserted into *Sac* I and *Xba* I of the modified pCAMBIA 1300 vector [29] to form a recombinant vector *35S*:*CpWRKY75*-*GFP*. *Agrobacterium* GV3101 carrying *35S*:*CpWRKY75*-*GFP* or *35S*:*GFP* were transferred into tobacco leaves using a disposable needleless syringe. After 36 h, GFP fluorescent was observed using confocal laser microscopy (Olympus, Tokyo, Japan).

### 2.5. Transcriptional Activity Analysis of CpWRKY75

The CDS of *CpWRKY75* was cloned into *Xma* I and *Sal* I of pGBKT7 to form a plasmid pGBKT7-*CpWRKY75*. The plasmid pGBKT7-*AtWRKY71* [31] and pGBKT7 were used as a positive control and negative control, respectively. The plasmids above were further introduced into AH109 yeast cells and cultured on SD/-Trp plates to select positive transformants, then the positive transformants were transported on SD/-His and SD/-His/X-α-gal plate for transcriptional activity assay.

### 2.6. Generation of CpWRKY75 Transgenic Arabidopsis Plants

The *35S*:*CpWRKY75*-*GFP* vector was transferred into Arabidopsis using the floral dip method [32]. Seeds harvested from T0 Arabidopsis were sown on solid MS containing 25 mg/L hygromycin, and hygromycin-resistant plants were selected for the further PCR identification. T3 generation Arabidopsis plants were used for phenotype observation.

### 2.7. Analysis of Transgenic Arabidopsis Plants under Normal Conditions

To observe the phenotype of transgenic plants under normal conditions, seeds of WT, EV and two independent *35S*:*CpWRKY75* lines (OE4 and OE17) were germinated on solid MS medium. After that, 10-day-old seedlings of Arabidopsis were transplanted into soil, and the phenotype was observed. The measurement of flowering time was performed as previously described [33].

## 3. Results

### 3.1. Isolation and Sequence Analysis of the CpWRKY75

**Figure 1:**
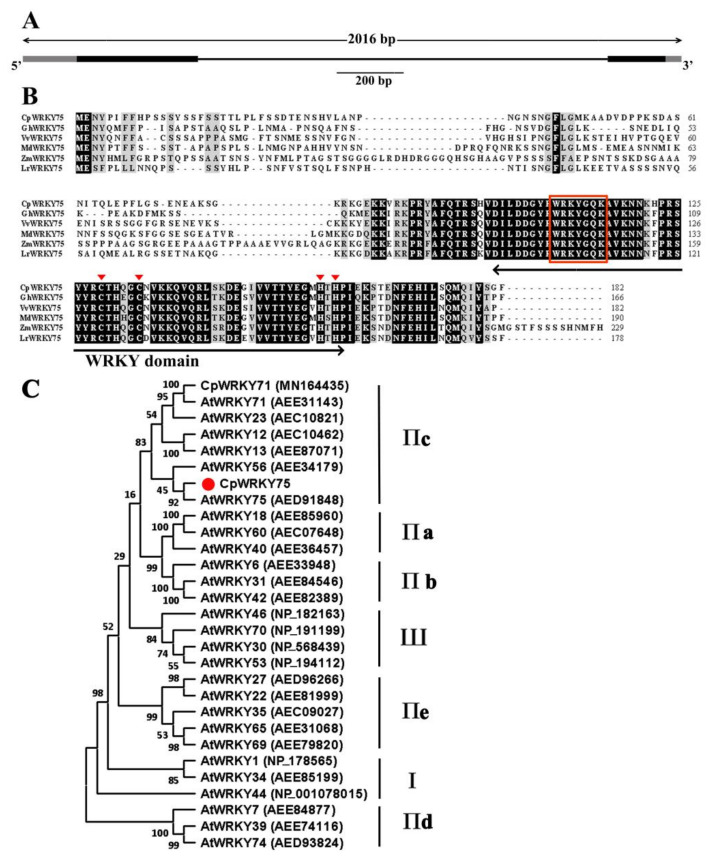
Sequence analysis of *CpWRKY75*. (**A**) Gene structure of *CpWRKY75*. Black boxes indicate exons, lines indicate introns, grey boxes indicate untranslated regions. (**B**) Multiple sequence alignment of CpWRKY75 and the other WRKY proteins. *Gossypium hirsutum* GhWRKY75 (XP_016725404.1), *Vitis vinifera* VvWRKY75 (XP_002274387.1), *Malus domestica* MdWRKY75 (XP_008389898.1), *Zea mays* ZmWRKY75 (PWZ27788.1) and *Lilium regale* LrWRKY75 (ART33473.1) are from GenBank. The WRKY domain is marked by a two-headed arrow. The WRKY motif and the zinc-finger motif are marked by red box and red triangle, respectively. (**C**) Phylogenetic tree of CpWRKY75 and WRKYs from other plants. The phylogenetic tree was constructed by MEGA 11 software with the neighbor-joining (NJ) method (1000 BootStrap). The solid red circle shape indicates the position of CpWRKY75.

The genomic DNA and full-length cDNA sequences of *CpWRKY75* were successfully cloned from wintersweet. Comparison of the *CpWRKY75* cDNA sequence with its genomic DNA sequence indicated that the *CpWRKY75* gene contained two exons and one intron, and the genomic DNA fragment was interrupted by a 1254 bp intron at the ‘GT’ and ‘AG’ sites (Figure 1A). The cDNA sequence of *CpWRKY75* contained a 549 bp coding region encoding a 182 amino acid protein. The putative molecular weight and theoretical isoelectric point (pI) of CpWRKY75 were 20.71 kDa and 9.24, respectively. CpWRKY75 exhibited high identities (62.37–79.23%) with its homologous sequences, including PmWRKY75 (XP_008222197), NtWRKY75 (XP_009615508), NnWRKY75 (XP_010249071) and CmWRKY75 (RWR87050). Multiple sequence alignment showed that CpWRKY75 protein contained a WRKY domain and a C2H2-type zinc finger motif (C-X4-C-X23-H-X1-H) (Figure 1B), indicating that CpWRKY75 is a member of group Ⅱ. A phylogenetic tree was constructed to evaluate the evolutionary relationship of CpWRKY75 with other WRKY proteins. Analysis of the phylogenetic tree showed that CpWRKY75 was clustered with group Ⅱc WRKY proteins and most closely associated with AtWRKY75 (Figure 1C). Interestingly, in this group AtWRKY75 [33], AtWRKY12, AtWRKY13 [25], AtWRKY71 [31] and CpWRKY71 [34] were proven to have a function in flowering-time regulation based on the previous studies, suggesting that CpWRKY75 might have a similar function in wintersweet.

### 3.2. CpWRKY75 Localizes to Nucleus and Exhibits no Transcriptional Activation Activity in Yeast

**Figure 2:**
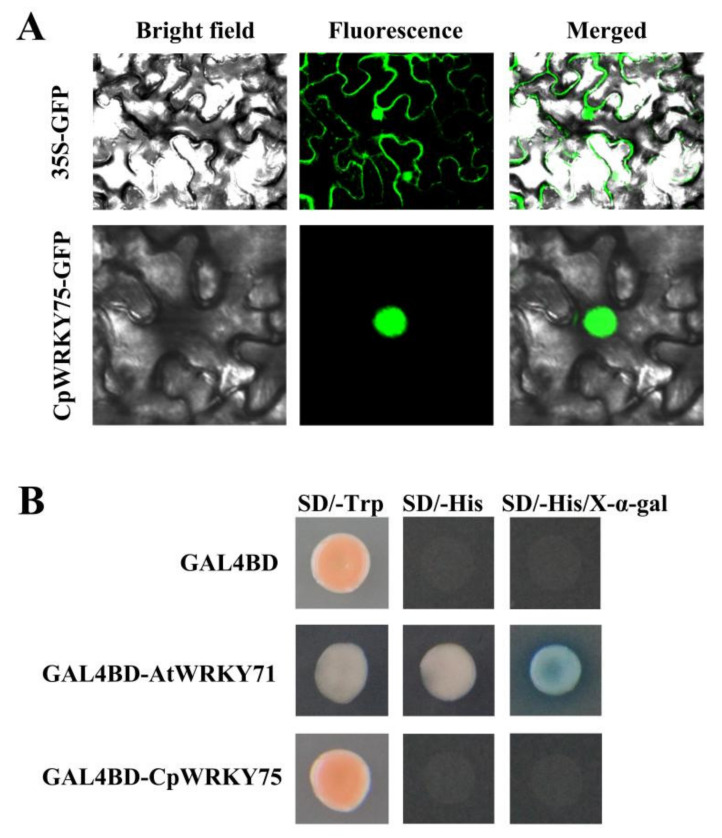
Nuclear localization and transactivation analysis of CpWRKY75. (**A**) Subcellular localization of CpWRKY75 in epidermal cells of tobacco leaf. (**B**) Transactivation analysis of CpWRKY75 was carried out using yeast AH109 cells.

The subcellular location of CpWRKY75 was predicted by the online software WoLF PSORT and ProtComp, and prediction results showed that CpWRKY75 was localized in the nucleus. In order to verify the prediction, the control vector *35S*:*GFP* and recombinant vector *35S*:*CpWRKY75*-*GFP* were transformed into tobacco epidermal cells using a needleless syringe. Confocal microscopic analysis showed that the green fluorescence of CpWRKY75-GFP was observed only in the nuclei of tobacco epidermal cells, whereas GFP control was distributed throughout the cytoplasm and nuclei (Figure 2A). In addition, in order to investigate the transcriptional activity of CpWRKY75, pGBKT7-*CpWRKY75*, negative control pGBKT7 and positive control pGBKT7-*AtWRKY71* were transferred into AH109 yeast cells. The results showed that all transformants could grow well on SD-Trp plates. The yeast cells harboring the positive control, which has been demonstrated to be a transcriptional activator [31], were able to grow on SD/-His plates and exhibited α-galactosidase activity on SD/-His/X-α-gal plates, whereas the yeast cells harboring pGBKT7-*CpWRKY75* and negative control could not (Figure 2B), indicating that CpWRKY75 is not a transcriptional activator. Collectively, our results indicated that CpWRKY75 is a nuclear-localized protein which has no transcriptional activation ability in yeast.

### 3.3. Expression Pattern of CpWRKY75 in Different Tissues

**Figure 3:**
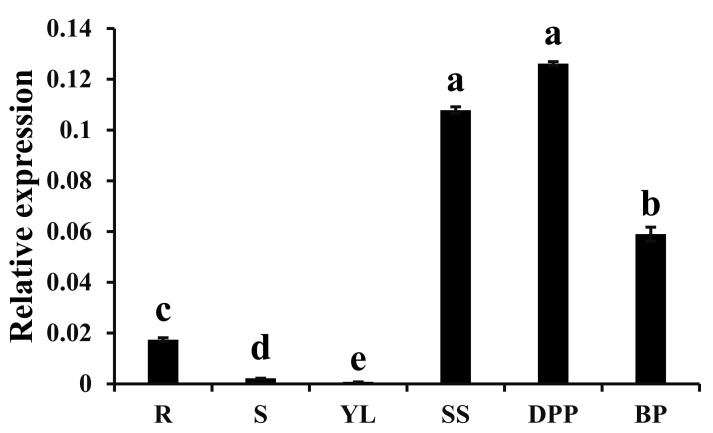
Expression profiles of *CpWRKY75* in different tissues. R: Roots; S: Stems; YL: Young leaves; SS: sprout stage; DPP: display-petal period; BP: bloom period. Lowercase letters shown above the columns indicate significant differences (one-way ANOVA, Duncan’s multiple range test, *p* < 0.01).

The expression pattern of *CpWRKY75* in wintersweet was determined by qRT-PCR. As shown in Figure 3, *CpWRKY75* expression was higher in flowers at different blooming stages than in vegetative tissues. In vegetative tissues, the highest transcript level of *CpWRKY75* was detected in roots; very low transcript levels of *CpWRKY75* were observed in young leaves and stems. In different flower blooming stages, *CpWRKY75* had a high expression in sprout stage and petal-display stage, and its expression slightly decreased in bloom stage.

### 3.4. Ectopic Overexpression of CpWRKY75 in Arabidopsis Caused Precocious Flowering

**Figure 4:**
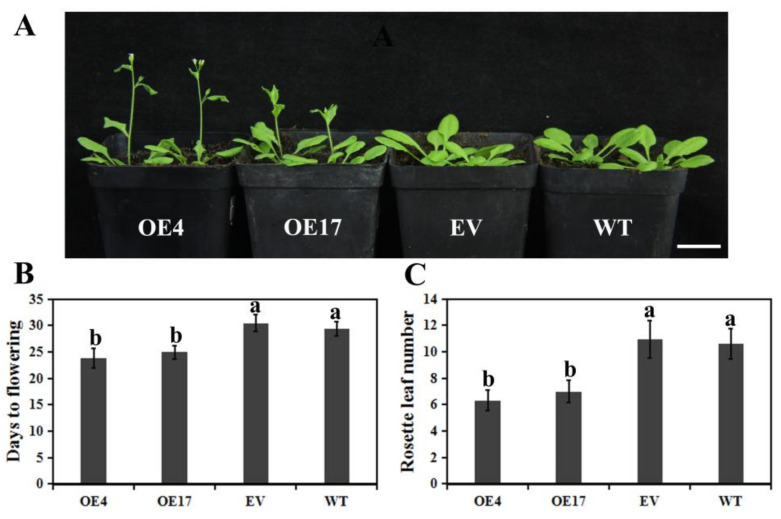
Early flowering phenomenon in *CpWRKY75* transgenic Arabidopsis. (**A**) The flowering phenotype of 24-day-old transgenic Arabidopsis, EV and WT. Bar denotes 2 cm. (**B**) Days to flowering for transgenic Arabidopsis, EV and WT. (**C**) The number of rosette leaves at flowering of transgenic Arabidopsis, EV and WT. Values are means ± SD (n = 20). Lowercase letters shown above the columns indicate significant differences (one-way ANOVA, Duncan’s multiple range test, *p* < 0.01).

To investigate the functions of *CpWRKY75*, transgenic Arabidopsis plants which over-express *CpWRKY75* were generated. Transgenic plants were confirmed using hygromycin selection and PCR analysis. *CpWRKY75* transcription levels in eighteen positive lines were detected by qRT-PCR. Two T3 overexpression (OE) lines (OE4 and OE17) with high *CpWRKY75* transcription levels were selected for phenotypic analysis. Under normal conditions, OE4 and OE17 exhibited a pronounced early flowering when compared with WT and EV plants (Figure 4A). OE4 and OE17 flowered at 23.8 and 24.9 days, while WT and EV needed 29.3 and 30.4 days to flowering (Figure 4B). In addition, the numbers of rosette leaves at flowering for OE4 and OE17 were 6.3 and 6.9, while for WT and EV plants the numbers were 10.9 and 10.6 (Figure 4C), which was consistent with the shortened flowering time of the transgenic Arabidopsis. These results imply that *CpWRKY75* may play a role in regulating flowering.

### 3.5. Expression Patterns of Flowering-Related Genes in CpWRKY75 Transgenic Plants

**Figure 5:**
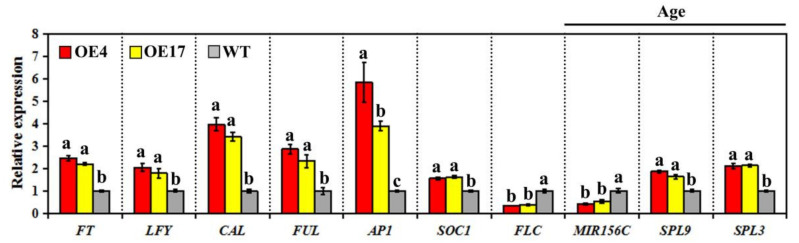
Transcript levels of flowering-related genes in *CpWRKY75* transgenic plants and WT plants. Fifteen-old-day plants were used for qRT-PCR analysis. Data represent means ± SD of three biological replicates. Lowercase letters shown above the columns indicate significant differences (one-way ANOVA, Duncan’s multiple range test, *p* < 0.01).

To explore the possible molecular mechanism by which *CpWRKY75* positively regulates flowering time, we investigated the regulating effects of *CpWRKY75* on the expression levels of flowering-related genes in the 15-day-old transgenic plants. The results showed that the expression levels of six genes involved in promoting flowering, including *FT*, *LFY*, *SOC1*, *AP1*, *CAL* and *FUL* were significantly increased, while that of the flowering-repressing gene *FLC* was significantly decreased in OE4 and OE17. However, the expression levels of vernalization (*VRN1* and *VRN2*), photoperiod (*CO* and *GI*), gibberellic acid (*RGA*, *GA20OX1* and *GAI*), and autonomous (*FLD*, *LD*, *FCA* and *FVE*) flowering pathway genes did not display any obvious differences between transgenic lines and control plants (Supplementary Figure S1). Interestingly, the transcript levels of the aging pathway genes, including *MIRNA156C*, *SPL3* and *SPL9*, were obviously altered in transgenic plants (Figure 5). These results indicate that *CpWRKY75* is likely to promote transgenic Arabidopsis flowering by regulating the expression of these flowering integrators and aging pathway genes.

## 4. Discussion

Flowering is of great importance for plants, especially for ornamental plants such as the wintersweet. For woody flowering plants, their long juvenile period makes it particularly difficult to promote their application and genetic improvement. In the past decades, advances in elucidating the mechanisms of Arabidopsis flowering made possible for researchers to carry out similar investigations in woody plants [35]. For instance, overexpression of *BpAP1* in birch caused early flowering and dwarfism [36]. Heterologous expression of loquat *EjLFY-1* resulted in precocious flowering in strawberry and its asexual progeny [6]. These studies show that transgenic technology has demonstrated tremendous potential to shorten the flowering time of woody flowering plants. However, a meaningful gene involved in regulating flowering time may play a critical role in this technology.

Substantial evidence has shown that WRKYs play an important role in regulating various aspects of plant growth and development including root development [37], seed germination [38], flowering [31,33], stem elongation, panicle development [22] and leaf senescence [19,20] as well as responses to various stresses [39,40]. However, research on WRKY genes has mainly focused on some model plants and crops, and the role of WRKY genes in wintersweet remains to be explored. In this study, we obtained a group IIc gene *CpWRKY75* from wintersweet and analyzed its characteristics. CpWRKY75 has one WRKY domain and its zinc finger motif is C2H2 type, suggesting that CpWRKY75 belongs to group II. Subcellular localization analysis showed that CpWRKY75 was located in the nucleus, which is consistent with the subcellular localization prediction, suggesting that CpWRKY75 might function in the nucleus. WRKY proteins can positively or negatively regulate the expression of downstream genes. In this study, a transactivation assay showed that CpWRKY75 has no transcriptional activation activity in yeast, which is similar to the results reported for ZmWRKY17 from *Zea mays* [41], GhWRKY42 from cotton [19], and BnWRKY184 from *Brassica napus* [42]. Activation of these WRKY proteins may require posttranslational modifications or need to be activated by some unknown upstream proteins [43]. Phylogenetic analysis showed that CpWRKY75 with group IIc WRKY proteins form a cluster and are closet to AtWRKY75, which was reported to be involved in regulating flowering. Mutation of *AtWRKY75* caused a delay in flowering, while overexpression of *AtWRKY75* dramatically promoted flowering [33]. Other group members such as AtWRKY12, AtWRKY13 [25], AtWRKY71 [31] CpWRKY71 [34] and BnWRKY184 [42] were also demonstrated to participate in flowering regulation. Thus, we speculate that *CpWRKY75* may share similar function with its orthologues, possibly in flowering regulation.

The expression patterns of genes often provide direction for their functional roles. For instance, *DOAP1* was expressed mainly in inflorescence apices and flowers at different developmental periods, and overexpression of *DOAP1* in Arabidopsis and orchids caused early flowering [44]. In our study, *CpWRKY75* had high transcript levels in different flower blooming stages, which indicates that *CpWRKY75* might participate in regulating flowering. To verify the role of *CpWRKY75*, *CpWRKY75* was transferred into Arabidopsis. The transgenic Arabidopsis plants showed an early flowering phenotype in comparison with WT and EV plants. All of these results suggest that *CpWRKY75* plays a role in regulating flowering.

Flowering is controlled by a complex genetic regulatory network. In Arabidopsis, there are five major genetically defined pathways which affect flowering, the vernalization, autonomous, photoperiod, gibberellin and aging pathways [45]. To understand how *CpWRKY75* regulates flowering, the transcripts of flowering-related genes involved in these five pathways were examined in transgenic Arabidopsis. Expression analysis showed that the expression of flowering-related genes from the vernalization, autonomous, photoperiod and gibberellin pathways were not obviously changed in transgenic Arabidopsis compared with WT. The expression levels of some aging pathways, however, were obviously altered in transgenic Arabidopsis compared with WT. The *MIRNA156C* gene plays an important role in the regulation of flowering time through the aging pathway. Decreased expression of *MIRNA156C* leads to increased expression of the target genes *SPL3* and *SPL9*, which in turn activates the transcription of the *FUL*, *SOC1*, *LFY* and *AP1* [46,47]. As excepted, the expression levels of *FUL*, *SOC1*, *LFY* and *AP1* were up-regulated in transgenic plants (Figure 5). These results indicate that CpWRKY75 might promote flowering by regulating the genes in the aging pathway. Moreover, WRKY regulates the expression of target genes by recognizing the W-box in the promoter sequence of these genes [4]. *FT*, *LFY*, *CAL*, *AP1* [31], *MIRNA156C* and *SPL3* (Supplementary File S1) exhibit W-boxes in their promoter sequences. Thus, there is a possibility that *CpWRKY75* promotes flowering in Arabidopsis by regulating the expression of these genes.

## 5. Conclusions

In conclusion, the wintersweet *CpWRKY75* gene belonging to group Πc of the WKRY superfamily was highly expressed in flowers at various bloom stages. Overexpression of *CpWRKY75* in Arabidopsis caused early flowering. The expression of flowering-related genes, including *FT*, *CAL*, *FLC*, *LFY*, *FUL*, *AP1*, *SOC1*, *MIRNA156C*, *SPL3* and *SPL9*, was significantly changed in transgenic Arabidopsis. Our study demonstrated that *CpWRKY75* could be a candidate gene for transgenic approaches to shortening the juvenile period in woody plants.

## Data Availability

Not applicable.

## Supplementary Materials

The following are available online at https://www.mdpi.com/article/10.3390/genes13010068/s1, Table S1: Primers used in this study. Figure S1: Transcript levels of vernalization, autonomous, photoperiod and gibberellin pathway genes in *CpWRKY75* transgenic plants and WT plants. File S1: The promoter sequences of *SPL3*, *MIRNA156C* and the distribution of W-boxes (TTGACT/C) in the promoters.
