# Supplementary material for: Overexpression of CpWRKY75 from Chimonanthus praecox Promotes Flowering Time in Transgenic Arabidopsis
Source: Genes (Basel). 2021 Dec 28;13(1):68. doi: 10.3390/genes13010068 (PMC8774968; doi:10.3390/genes13010068)
Supplement: Supplementary file 1 [file genes-13-00068-s001.zip › genes-1444640-supplementary.pdf]

Supplementary Table S1. Primers used in this study

Primer name	Primer sequence (5′-3′)
	Clone of *CpWRKY71*
CpWRKY75-F	CTTTCCTTCTCCCATTTCTT
CpWRKY75-R	GGACATAAGGTAAACAGTCAGCT
	Subcellular localization
CpWRKY75-Sac I-F	GCGAGCTCATGGAAAACTACCCTATATTCTT
CpWRKY75-Xba I-R	GCTCTAGAAAATCCAGAATAGATCTGCAT
	Transactivation assay
CpWRKY75-Xma I-F	TCCCCCCGGGGATGGAAAACTACCCTATATTCTTCC
CpWRKY75-Sal I-R	GCGTCGACAAATCCAGAATAGATCTGCATCTG
	qRT-PCR for wintersweet
CpActin-F	AGGCTAAGATTCAAGACAAGG
CpActin-R	TTGGTCGCAGCTGATTGCTGTG
CpTublin-F	GTGCATCTCTATCCACATCG
CpTublin-R	CAAGCTTCCTTATGCGATCC
qCpWRKY75-F	CACACGTTCTCGCCAATCCT
qCpWRKY75-R	AGGGTTCCAGTTGGGTGATG
	qRT-PCR for Arabidopsis
qActin-F	CTTCGTCTTCCACTTCAG
qActin-R	ATCATACCAGTCTCAACAC
qFT-F	TTCCAAGTCCTAGCAACCCTCACC
qFT-R	TTCTTCCTCCGCAGCCACTCTC
qFUL-F	GCCTCAATACTGCGTAACCTCC
qFUL-R	GGTAGGACGTAACATCCAAGCC
qCAL-F	AAGAAGACCAAACGGCGATG
qCAL-R	GGCGTAACAGCCAAGGTAATTG
qLFY-F	CTCTATTTGGTATGTTCCAACAAAG
qLFY-R	CTAATACCGCCAACTAAAGCC
qSOC1-F	AGCTGCAGAAAACGAGAAGC
qSOC1-R	TGAAGAACAAGGTAACCCAATG
qAP1-F	CATGGGTGGTCTGTATCAAGAAGAT
qAP1-R	CATGCGGCGAAGCAGCCAAGGTT
qFLC-F	AGCCAAGAAGACCGAACTCA
qFLC-R	TTTGTCCAGCAGGTGACATC
qFVE-F	GAGACTACTGGTGGAGGTGGAAC
qFVE-R	TGAACCAGAACCATGTACCCCTAAC
qLD-F	GGATTGAATCGTCACAGGGTCC
qLD-R	CCATCTGTACTTTCCTGCGTTCT
qFLD-F	ACAATGCCACCCACTGACGC
qFLD-R	GCAGGGTATCGCCTTGTTGTG
qRGA-F	TACATCGACTTCGACGGGTA
qRGA-R	GTTGTCGTCACCGTCGTTC
qGAI-F	AACTCGGCATGTTGTCCTG
qGAI-R	AAAGCGCGTGAACGAGAC
qGA20OX1-F	GTCAATCACGGCATCAGCG
qGA20OX1-R	CCAACGCATCGCAGAAGTAATC
qSPL3-F	CTCATGTTCGGATCTCTGGTCTT
qSPL3-R	TTTCCGCCTTCTCTCGTTGTG
qGI-F	AATTCAGCACGCGCCTATTG
qGI-R	GTTGCTTCTGCTGCAGGAACTT
qSPL9-F	CCAAGGTGCCAAGTGGAAGG
qSPL9-R	AAGCGAAGGTGCGATCCTCCCGT
qCO-F	TTGCTTCGTGGCTGTTCCC
qCO-R	TCCCTTTGGGCGTTCTTGG
qCRY1-F	CTAGGTGGTGGCTCAAGAACAGT
qCRY1-R	CGTTGAATGATCGAACCGCTAT
qCRY2-F	ACCCAGAAGGTGAGTACATAAGG
qCRY2-R	GCCGAAGGTACTTGTTGGTCATT
qVRN1-F	TGTTGACCGTTACTCCATTCGC
qVRN1-R	TGTGAGCGGAATCCATGAGAC
qVRN2-F	AAGCGTAGACAAAGAGGTGGCA

qVRN2-R	GGGCTATGGCTGGTGGAATG

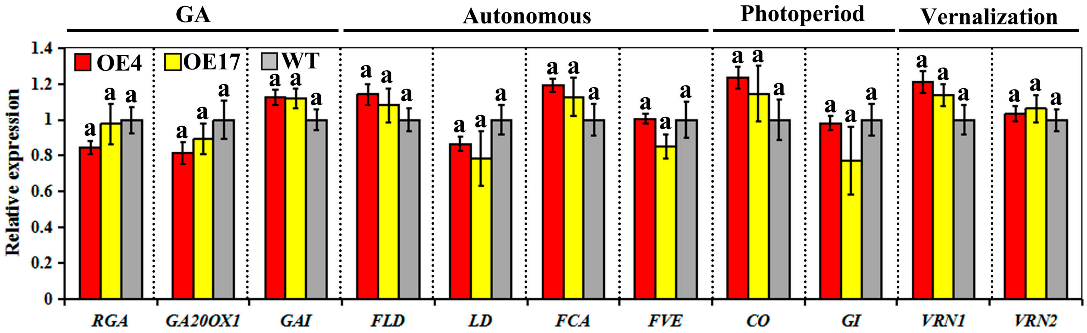

Supplementary Figure S1. Transcript levels of vernalization, autonomous, photoperiod and gibberellin pathway genes in *CpWRKY75* transgenic plants and WT plants. Fifteen-old-day plants were used for the qRT-PCR analysis. Data represent means ± SD of three biological replicates. Lowercase letters shown above the columns indicate significant differences (one-way ANOVA, Duncan's multiple range test, $p < 0.01$).

Supplementary File S1. The promoter sequences of *SPL3*, *MIRNA156C* and the distribution of W-boxes (TTGACT/C) in the promoters. The red highlighting indicates the W-box sequence.

Promoter of *MIRNA156C*
TACCTGGTTTCTTACTCGTTAACACGTGTCCAATTGAATTAATTCTTAAAATTGTGAAA-
TAATTTTAAAGATTAAGTTACAATTGATATTTGAGGTTCCAA-
TATGTATTCCTCCCTCAAAACTTTAATATATCTGTGTTAGTTTCACCTAGAC-
CTTACTTCTCTTTACTTCTCTTACTATTAATTATTACTTCTCTTACAGCTATTTTCACATA-
TATATAGAAAGTTTAAAACCTGTATACACGTTTATTTAAACTAGTATTGTTTTAGGTGAAAA-
TAGCAAAAAGGACAAAGTATCTGGGTGAAATTGGTGGTATATGAAAATTTCAAAAGGTCGAC-
CTGACATTTTTGTATTATATGTGTTTGGTAAAATCACAAAAGATGGAAAACCTATGAAATAA-
TATTTCCAAAGAAATGTGTGATCTTTTTACAAAGATTAGAAGATGACCTATAGGTGG-
CATCAACATGTTATATGATCAATTGGTAACTTTTAAAACTATCATGGTTTTAGTAAATCAATT-
AGAAAGAGTTAAACACCAAATAGTTACAATTTGGTCAATTTTATTTGTTCGGTTAATTT-
GTGTTTGTTTGCTGTAGTAGAAGTCTTTGATAAAGATTTATTGTATTTGTATTTTAAAATTAT-
TATAATATTAAAAGAGCTGATTCCCAAAAAATAATATTTACCACTCCCATCGTGAAAGAC-
CAAAATTCATTTCTCAAAATTCTAGATCATTTTCTAGGCTTGTCGTT-
GCCGTTTATAGGTTATGAGATTGACCACTATAATCTGCAATGGTACTGTT-
GAATCCAATCCGTTCCATTCAAATGTCTCTCTCTTCAGACATCTGTCCCATTGCATGTAA-
GAGGGAGAGAGATAAGAAACAGACAAAAGCCAAAAAAAAAAAAAACAGAGATAA-
GACTCTTCTTGAAGAGAGTGAGAGACAGAGAGAGATAAGGTTTTTT-
GTTTCCTTCTTTAACAGAT-
TCTTCCTCTCTTCTCCTCTCCTCTTATTAATCTAATCCTCCTCCCCGAATATTTCTCTGCCTT-
TAGTTCTTTCTTTTTTGGTAATATATTTATTTTTCGTTACGATTTGGTCAAAACCCTAGAT-
TTGTTTTCCAAAAGCATATCTGAAAATGAAGGACAACTTTCCTCTTCTCCTTCGGTTATAAA-
TATTCTCTCCGGTTTTGCTTGTTTAACCTAAAAGCCTCAGATCTAACTCCAACAC-

CTTCAAAGTCTGCCTCCTTTCCAATCTTCTTTCTTCTGTTCGATCTCTAATCTCAGAATTT-

GTGTCGGTAAGGTAAAGGTGATAATGAGTGATGACTGATGAGGGAGTTTTGGGACAAATTTTAAGAGAAA

Promoter of *SPL3*

TGAGACCTGAGTACAAGATCGAAAAGAAAATATCTCGAATAACAGAGTTGTGTTTGCTGTTTT-
TACCATTTAATTATGCTAATATCTTTGTTTTACATAATACCAATT-
GAAAAATGAATACATAATTCAAGTTTGGACCATTCTTGTATATCCACTTTT-
GGCTTCGTGGCCCCGATAGCTAATTTGATTGACCTTATCATCTACACCAAACAA-
GAAATTAAATTGTCAAATTAATTTAGTGGTGGCCCTATACTATAAAATCGTAAACAC-
TTCTTTTTGAAACTAAATATAAACCTTACGACACATTTATGTGCAAATTAGTG-
GACATATTCCTCAGTAATAACGTTTTTACAAACCTACAGTTGGATAAATTATTGAGTCAC-
TCCATGATTAAACCAACAACATAAGAGAAAAATAAAATAGTACACTACGAATGATCTTTTAAA-
TATGTTATTCTTTTTTGATACAGAAACTTTAACTACCAAAAAAAAAAAAGCGAATGAT-
TCATAAAAACGCAATGTGGTATGTTTGGTCTGTTTTTTAGTTCGAATCTCAACTTCTTGTTAC-
CACAAAAATAAAACATTACACAATGTAAATTTACTGTTCAGTCTTGCTTTTCCAATCAC-
TACATTAGCACATATTCTTCACATGTATTTTCCACTAATCTCGCGACAAAATGTTCACAG-
TGTTTCTCAGTTTCTCTCTCTCAATATTTTCTTGTCAAACAACATGCATATTTGAACCCG-
CATGGTGAAATAGCATCGGCATTAACATGATCAACCACAAAATAAAC-
CGGGGTGGTGTCATAGCATCGACATTAACTTAAAACTACAAAA-
TAGAGCTGGGTCCTCGTCAGGTTTTAAGGATACATATGTATGCATGTATGCATATACAAAA-
TAATAGAGCACTAAAGCTTGTTCGTACTCTTTTTTACACAACATGCTAATAATTTTTATACCT-
TAGGAGTGAAATTTTAGTGGTCCAACAACTTAAGCACCTAAGATCCTTCCCCACTTTTGTTT-
GGGGCTCCTTTATATCTCTTCCAATGGTTACTTTTACTTCCTCTTCTTCTCTTCTT-
GTTCCATCTTTCATGTGAGAGAGAGAGTTGAATTTTGCAG
